# Fueling bone loss: the immunometabolic reprogramming of the bone microenvironment in diabetic osteoporosis

**DOI:** 10.3389/fimmu.2026.1850044

**Published:** 2026-06-30

**Authors:** Yunfang Wang, Jinwen Han, Zheng Wang, Gai Zhang, Ruilan Niu, Yaqing Wei, Kang Yi, Jinxing Quan

**Affiliations:** 1 Department of Endocrinology, Gansu Provincial Hospital, Lanzhou, Gansu, China; 2 The First Clinical Medical College of Lanzhou University, Lanzhou, Gansu, China; 3 Department of Endocrinology, Key Laboratory of Endocrine and Metabolic Diseases of Gansu Province, Lanzhou, Gansu, China; 4 PET/CT Center, Gansu Provincial Hospital, Lanzhou, Gansu, China; 5 The first Clinical Medical College of Gansu University of Chinese Medicine (Gansu Provincial Hospital), Lanzhou, Gansu, China; 6 Department of Geriatric, Gansu Provincial Central Hospital, Lanzhou, Gansu, China; 7 Department of Geriatrics, Gangu County People’s Hospital, Tianshui, Gansu, China; 8 Department of Cardiovascular Surgery, Gansu Provincial Hospital, Lanzhou, China; 9 Gansu International Scientific and Technological Cooperation Base of Diagnosis and Treatment of Congenital Heart Disease, Lanzhou, China

**Keywords:** bone microenvironment, bone remodeling, diabetic osteoporosis, immunometabolism, macrophage, metabolic reprogramming, Th17/Treg balance

## Abstract

Diabetic osteoporosis (DOP) is a serious skeletal complication of type 2 diabetes mellitus (T2DM), characterized by deteriorated bone microarchitecture and elevated fracture risk independent of reduced bone mineral density. Emerging evidence indicates that immunometabolic reprogramming of bone marrow immune cells serves as a core driver of DOP pathogenesis. This review focuses on macrophages and CD4+ Th17/Treg cells, the pivotal immune subsets mediating skeletal immune-metabolic homeostasis. We systematically elaborate how hyperglycemia triggers glycolytic predominance, HIF-1α stabilization, and succinate-SUCNR1 axis activation in macrophages, alongside mTORC1-HIF-1α-dependent Th17/Treg imbalance. Bidirectional immunometabolic crosstalk between these immune cells disrupts the RANKL/OPG and Wnt/β-catenin signaling pathways, thereby disturbing bone formation and resorption. Furthermore, we summarize current immunometabolic-targeted modulators for DOP, stratifying their preclinical and clinical evidence, skeletal benefits, and safety limitations. We also highlight existing clinical diagnostic defects and translational bottlenecks in DOP research. Finally, we prospect emerging research directions including spatial immunometabolic profiling, gut-immune-bone axis regulation, and artificial intelligence-assisted precision intervention, aiming to provide mechanistic insights and novel translational strategies for DOP targeted therapy.

## Graphical Abstract

**Figure:**
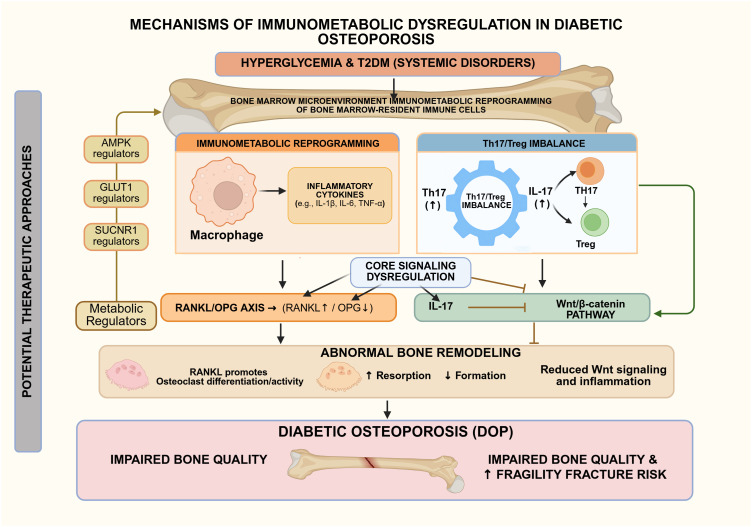
Schematic overview of the core immunometabolic mechanisms underlying diabetic osteoporosis (DOP) and potential therapeutic strategies. Chronic hyperglycemia in type 2 diabetes (T2DM) triggers immunometabolic reprogramming of bone marrow-resident immune cells, including macrophage M1 polarization (driven by the succinate-SUCNR1 axis and HIF-1α stabilization) and Th17/Treg imbalance (mediated by the mTORC1-HIF-1α axis). These dysregulations lead to excessive osteoclast activation and impaired osteoblastogenesis via disruption of the RANKL/OPG axis and Wnt/β-catenin signaling pathway, ultimately resulting in bone quality impairment and increased fragility fracture risk. Targeting key immunometabolic checkpoints (e.g., AMPK, GLUT1, SUCNR1) may restore immune-metabolic homeostasis and mitigate skeletal damage in DOP. Abbreviations: HIF-1α, hypoxia-inducible factor 1α; mTORC1, mechanistic target of rapamycin complex 1; OPG, osteoprotegerin; OXPHOS, oxidative phosphorylation; RANKL, receptor activator of nuclear factor κB ligand; SOST, sclerostin; SUCNR1, succinate receptor 1; Th17, T helper 17 cells; Treg, regulatory T cells.

## 1 Introduction

Type 2 diabetes mellitus (T2DM) and osteoporosis are widespread chronic metabolic disorders, whose pathological intersection leads to diabetic osteoporosis (DOP), a critical public health burden associated with increased fragility fractures (1, 2). Notably, T2DM patients frequently exhibit normal or elevated BMD but significantly higher fracture risk than non-diabetic individuals, indicating that bone microstructural deterioration rather than bone mass loss dominates DOP pathogenesis (3, 4). Multiple pathological factors, including chronic hyperglycemia, glycemic fluctuation, oxidative stress, advanced glycation end products deposition, and bone marrow microenvironment dysfunction, jointly disrupt bone remodeling (5–8). In addition, dysregulated autophagy (especially mitophagy), vitamin D receptor (VDR) gene polymorphisms, abnormal secretion of adipokines such as leptin, and impaired osteogenic differentiation of bone marrow mesenchymal stem cells (BMSCs) jointly form a complex pathophysiological network of DOP (9–13).

Clinically, conventional hypoglycemic and anti-osteoporotic agents fail to achieve sufficient efficacy and inconsistent skeletal safety in DOP treatment, resulting in prominent therapeutic dilemmas (14–16). Existing reviews primarily summarize macroscopic metabolic disorders and intrinsic bone cell dysfunction, while rarely systematically elucidating immunometabolic crosstalk within the bone marrow niche. The immune-metabolic reprogramming of resident immune cells, serving as the key bridge between systemic diabetes and local skeletal damage, remains insufficiently integrated and interpreted in current literature.

Addressing this critical research gap, the present review centers on bone marrow immunometabolic remodeling during DOP pathogenesis. This work systematically delineates metabolic stress-induced rewiring of bioenergetic profiles and immune polarization in macrophages and CD4^+^T cells, while clarifying how bidirectional crosstalk between these immune subsets disrupts physiological bone remodeling. Integrating stratified preclinical and clinical evidence regarding immunometabolic regulators, this article characterizes the translational feasibility and latent safety risks of DOP-targeted immunometabolic therapies. Current diagnostic shortcomings and translational bottlenecks in DOP clinical transformation are also highlighted, with emerging innovative research directions prospected to facilitate the development of precision immunometabolic intervention strategies for DOP.

## 2 Macrophage metabolic reprogramming: the engine of bone resorption in diabetes

Bone marrow macrophages are the cornerstone of osteoclastogenesis and skeletal immune homeostasis, and metabolic plasticity reshapes macrophage functional phenotypes within the diabetic bone microenvironment (17). Under physiological conditions, macrophages maintain a quiescent or M2 anti-inflammatory phenotype, relying on OXPHOS and fatty acid oxidation (FAO) for energy supply to support bone protection (18, 19). In this homeostatic state, FAO sustains stable mitochondrial function, restricts pro-inflammatory transcription programs, and enables M2 macrophages to secrete trophic factors that stabilize osteoblast activity and inhibit excessive osteoclast formation. Unfortunately, the immune response is disrupted in a high glucose (HG) microenvironment (20–22). With dysfunction of mitochondria and endoplasmic reticulum under such condition (23, 24), macrophages rapidly transition from a resting state to a highly active state, exhibiting a pronounced increase in the production of proinflammatory M1-secreted cytokines (25–27), which impairs tissue repair and bone healing. In diabetes, chronic glucotoxicity rewires macrophage metabolism, triggering a pro-inflammatory M1-like shift that acts as the core engine of pathological bone resorption (28–30) (**Table 1**).

**Table 1:** Key immunometabolic abnormalities in the diabetic bone marrow niche.

Cellular player	Metabolic reprogramming	Key signaling hubs	Major effector molecules	Impact on bone homeostasis	References
Macrophages	Switch from OXPHOS/FAO to aerobic glycolysis; mitochondrial dysfunction; succinate accumulation	HIF-1α, SUCNR1, NLRP3	IL-1β, IL-6, TNF-α, lactate	Promotes M1 polarization; enhances osteoclast differentiation and activity; acidic microenvironment accelerates bone matrix degradation	(24, 30, 51, 56, 62)
Th17 Cells	Enhanced GLUT1-dependent glycolysis; reduced mitochondrial respiration	mTORC1-HIF-1α, RORγt	IL-17A	Upregulates RANKL and suppresses OPG; stimulates sclerostin secretion; inhibits Wnt/β-catenin-driven bone formation	(88, 99, 103, 106)
Treg Cells	Metabolic exhaustion; impaired mitochondrial function; glucose competition	AMPK, FOXP3 instability	Loss of TGF-β, IL-10; conversion to pro-inflammatory ex-Tregs	Diminished immunosuppression; unchecked inflammation; accelerated osteoclastogenesis	(85, 117, 119, 122)
Osteocytes	Oxidative stress-mediated dysfunction	Wnt/β-catenin inhibition	Sclerostin (SOST)	Blocks osteoblast proliferation and differentiation; suppresses bone matrix synthesis	(106, 107, 110)
Osteoclast Precursors	Metabolically activated by inflammatory signals	RANKL-RANK, TNF-α synergism	Cathepsin K, V-ATPase, ClC-7	Excessive bone resorption; degradation of hydroxyapatite and type I collagen	(56–59)

AMPK, Adenosine Monophosphate-Activated Protein Kinase; FAO, Fatty Acid Oxidation; FOXP3, Forkhead Box P3; GLUT1, Glucose Transporter 1; HIF-1α, Hypoxia-Inducible Factor-1α; IL-10, Interleukin-10; IL-17A, Interleukin-17A; IL-1β, Interleukin-1β; IL-6, Interleukin-6; mTORC1, Mechanistic Target of Rapamycin Complex 1; NLRP3, NOD-Like Receptor Pyrin Domain-Containing Protein 3; OPG, Osteoprotegerin; OXPHOS, Oxidative Phosphorylation; RANK, Receptor Activator of Nuclear Factor-κB; RANKL, Receptor Activator of Nuclear Factor-κB Ligand; RORγt, Retinoic Acid-Related Orphan Receptor Gamma T; SOST, Sclerostin; SUCNR1, Succinate Receptor 1; TGF-β, Transforming Growth Factor-β; TNF-α, Tumor Necrosis Factor-α; V-ATPase, Vacuolar Adenosine Triphosphatase.

### 2.1 The shift from OXPHOS to glycolysis: the warburg-like effect in diabetic macrophages

Under physiological conditions, quiescent bone marrow macrophages rely on OXPHOS and FAO to sustain anti-inflammatory and bone-protective phenotypes (31–33). However, persistent HG acts as a decisive metabolic switch, upregulating macrophage membrane GLUT1 expression to enhance glucose influx and trigger a Warburg-like metabolic shift toward aerobic glycolysis (34, 35). This metabolic reprogramming inhibits protective FAO/OXPHOS metabolism and drives macrophage pro-inflammatory and pro-osteoclastogenic polarization (36–38). Enhanced glycolytic flux diverts glucose metabolites into the PPP, boosting NADPH generation and subsequent NADPH oxidase-dependent ROS production. Continuous ROS accumulation prevents HIF-1α degradation and facilitates HIF-1α nuclear translocation and transcriptional activation (30, 39–42). At the organismal level, this metabolic switch has been validated in diabetic mouse models, where bone marrow macrophage-specific glycolytic hyperactivation consistently correlates with reduced trabecular bone volume fraction, increased trabecular separation, and elevated osteoclast number in femoral metaphyses (43–45). Single-cell RNA sequencing has confirmed that glycolytic gene signatures are significantly enriched in bone marrow macrophages under diabetic conditions, which is directly correlated with enhanced osteoclast differentiation and activity (46, 47).

### 2.2 HIF-1α as a molecular bridge to osteoclastogenesis

Stabilized HIF-1α acts as the core downstream effector of HG-triggered glycolytic dysfunction and functions as a vital immunometabolic checkpoint bridging metabolic reprogramming and osteoclastogenesis. Hyperglycemia-triggered mTORC1 hyperactivation further promotes hypoxia-independent HIF-1α protein accumulation and nuclear translocation, connecting systemic glucotoxicity and local macrophage inflammatory activation (48). Upon activation, HIF-1α directly initiates the transcription of pro-inflammatory genes (IL-1β, IL-6, TNF-α), driving macrophages toward a pro-osteoclastogenic phenotype (28, 49–51).

Within the bone marrow niche, these cytokines function as potent paracrine signals that amplify osteoclastogenesis. TNF-α synergizes with RANKL signaling, significantly lowering the activation threshold of osteoclast precursor cells and promoting their differentiation into mature bone-resorbing osteoclasts (52–55). Moreover, glycolysis-derived lactate accumulates extracellularly to induce local acidosis, which facilitates hydroxyapatite dissolution, activates osteoclastic V−ATPase and ClC−7, and optimizes catalytic conditions for bone-degrading cathepsin K, ultimately aggravating bone matrix degradation (56–59). Genetic and pharmacological inhibition of macrophage HIF-1α in diabetic rodent models has been demonstrated to reverse glycolytic predominance, reduce pro-inflammatory cytokine release, and substantially restore trabecular microarchitecture while suppressing excessive osteoclastic bone resorption, confirming the causal link between HIF-1α-dependent immunometabolic dysfunction and DOP bone loss (60, 61). Consistent with these mechanistic insights, emerging clinical evidence has linked increased HIF−1α activity in circulating immune cells to worse skeletal outcomes in diabetes, further supporting the translational relevance of HIF−1α−driven immunometabolic reprogramming in diabetic bone fragility.

### 2.3 The succinate-SUCNR1 signaling axis: mitochondrial dysfunction link to skeletal fragility

Beyond glycolytic reprogramming, disrupted mitochondrial metabolism represents another key driver of macrophage dysfunction in DOP. In the HG microenvironment, glycolytic overload disrupts TCA cycle homeostasis in macrophages, leading to mitochondrial dysfunction and massive intracellular succinate accumulation (24, 62). Macrophages secrete accumulated succinate extracellularly as a metabolic alarmin to initiate persistent inflammatory cascades (63, 64).

Extracellular succinate binds to the macrophage and osteoclast precursor receptor SUCNR1 (GPR91), forming a positive feedback loop upstream of the established HG-GLUT1-glycolysis-HIF-1α axis. Succinate-SUCNR1 ligation further activates mTORC1 signaling, potentiating sustained HIF-1α stabilization and glycolytic activation, thereby continuously amplifying macrophage pro-inflammatory polarization and bone catabolism (65, 66). Functionally, this signaling cascade drives osteoclast precursor fusion and hyperactivation, leading to increased bone resorption depth and impaired bone microarchitecture in diabetic murine models (4, 67). This autocrine and paracrine loop further enhances HIF-1α activation and pro-inflammatory cytokine secretion, while directly promoting the fusion of mononuclear pre-osteoclasts into multinucleated hyperactive osteoclasts (60, 68–71). Preclinical studies have demonstrated that targeted inhibition of the succinate-SUCNR1 axis significantly reverses bone loss in diabetic models by suppressing macrophage metabolic reprogramming and excessive osteoclastogenesis, identifying this pathway as a novel therapeutic target for DOP (65, 72, 73).

## 3 The Th17/Treg imbalance: fueling the inflammatory bone loss

CD4^+^ T lymphocyte subsets are pivotal regulators of skeletal immune homeostasis, with Th17 cells driving pro-inflammatory and pro-osteoclastogenic responses, and T regulatory (Treg) cells exerting anti-inflammatory and bone-protective effects (74, 75). The dynamic balance between Th17 pro-catabolic and Treg anabolic-protective functions strictly governs physiological bone remodeling. In diabetes, nutrient overload and metabolic stress disrupt the metabolic programming of CD4^+^T cells, triggering severe Th17 hyperactivation accompanied by Treg functional exhaustion. This bidirectional defective interplay synergistically amplifies niche inflammation, accelerates osteoclast overactivation, and ultimately induces persistent inflammatory bone loss (76–78) (**Table 1**).

### 3.1 Nutrient-sensing pathways and T cell fate determination

The differentiation of CD4^+^T cells into specialized effector lineages is fundamentally governed by their metabolic status, with distinct metabolic programs dictating subset-specific functions. Under physiological conditions, Treg cells rely on FAO and OXPHOS for energy supply, maintaining FOXP3 expression and immunosuppressive activity to protect bone homeostasis (79, 80). In contrast, Th17 cells are highly dependent on GLUT1-mediated glycolysis, which fuels the synthesis of pro-inflammatory cytokines (80–82).

In the hyper-nutrient diabetic milieu, aberrant mTORC1-HIF-1α axis activation serves as the master switch driving CD4^+^T cell metabolic reprogramming. Mechanistically, hyperglycemia-induced mTORC1 activation serves as the core upstream sensor that rewires CD4^+^T cell bioenergetics: it suppresses mitochondrial FAO and OXPHOS while transcriptionally upregulating GLUT1 and key glycolytic enzymes (83, 84). This metabolic reprogramming exerts opposing effects on two CD4^+^ subsets simultaneously: it selectively promotes glycolysis-dependent Th17 differentiation and pro-osteoclastogenic priming via RORγt upregulation, whereas impaired mitochondrial metabolism destabilizes FOXP3 and induces Treg functional deficiency. The metabolic competition for glucose and mitochondrial resources further exacerbates the divergent fate of Th17 and Treg cells (85–87). *In vivo*, diabetic mouse models with mTORC1 inhibition exhibit restored Treg FAO capacity, corrected Th17/Treg ratio, and improved trabecular bone mass, verifying that mTORC1-HIF-1α-mediated metabolic switching directly determines bone immune homeostasis (88–91). Hyperglycemia activates mTORC1, which upregulates HIF-1α expression and glycolytic gene transcription, selectively promoting the expression of RORγt (the master transcription factor for Th17 cells) while antagonizing FOXP3-driven Treg differentiation (88, 92–94). In the diabetic bone marrow, Th17 cells undergo a clear metabolic switch from OXPHOS to glycolysis, accompanied by suppressed mitochondrial respiration. In contrast, Treg cells exhibit compromised metabolic and immunosuppressive function due to impaired mitochondrial activity and insufficient glycolysis (76, 95–97). Collectively, this reciprocal metabolic disorder results in concurrent Th17 pro-inflammatory overactivation and Treg bone-protective failure, forming a dual pathological mechanism that disrupts bone immune homeostasis.

### 3.2 IL-17: the molecular bridge between hyperglycemia and bone remodeling disorder

The expansion of Th17 cells in diabetes leads to a surge in IL-17A secretion, both systemically and within the bone marrow niche, serving as the key molecular mediator linking immunometabolic imbalance to skeletal damage (98–101). Notably, concurrent Treg dysfunction exacerbates the pro-osteoporotic effect of Th17-derived IL-17: impaired Treg cells fail to secrete sufficient anti-inflammatory TGF-β and IL-10 to counteract IL-17-mediated niche inflammation (102). IL-17 disrupts bone homeostasis through two complementary mechanisms: first, it directly acts on bone marrow stromal cells (BMSCs) and osteoblasts to upregulate RANKL expression and suppress OPG secretion, tilting the RANKL/OPG ratio toward osteoclastogenesis (103–105). Taken together, excessive IL-17-driven bone catabolism and lost Treg-mediated immune tolerance jointly disrupt bone remodeling equilibrium, accelerating DOP progression.

Second, IL-17 synergizes with high-glucose-induced oxidative stress to promote sclerostin (SOST) secretion from osteocytes. Sclerostin is a potent antagonist of the Wnt/β-catenin signaling pathway, which is essential for osteoblast proliferation, differentiation, and bone formation (106, 107). Inhibition of Wnt/β-catenin signaling leads to a profound suppression of osteoblastogenesis and bone matrix synthesis, exacerbating the imbalance between bone resorption and formation (108–110). Consistently, diabetic mice with IL-17A knockout exhibit preserved Wnt/β-catenin activity, elevated bone formation rate, and reduced osteoclastic activity, confirming that Th17-derived IL-17 is indispensable for immunometabolism-coupled bone formation suppression in DOP models (111–113). Clinical evidence supports that elevated serum IL−17 levels are positively associated with increased fracture risk in type 2 diabetes patients with impaired bone quality, independent of bone mineral density, further highlighting the pathological contribution of IL−17 to diabetic skeletal fragility (111, 113).

### 3.3 Treg dysfunction and the loss of skeletal protection

In healthy individuals, Treg cells secrete anti-inflammatory cytokines (TGF-β, IL-10) that inhibit osteoclast differentiation and promote osteoblast function, maintaining skeletal immune tolerance (114–116). However, chronic hyperglycemia induces Treg metabolic exhaustion, abolishes immune suppressive capacity, and triggers aberrant phenotypic conversion (117, 118).

**Figure 1:**
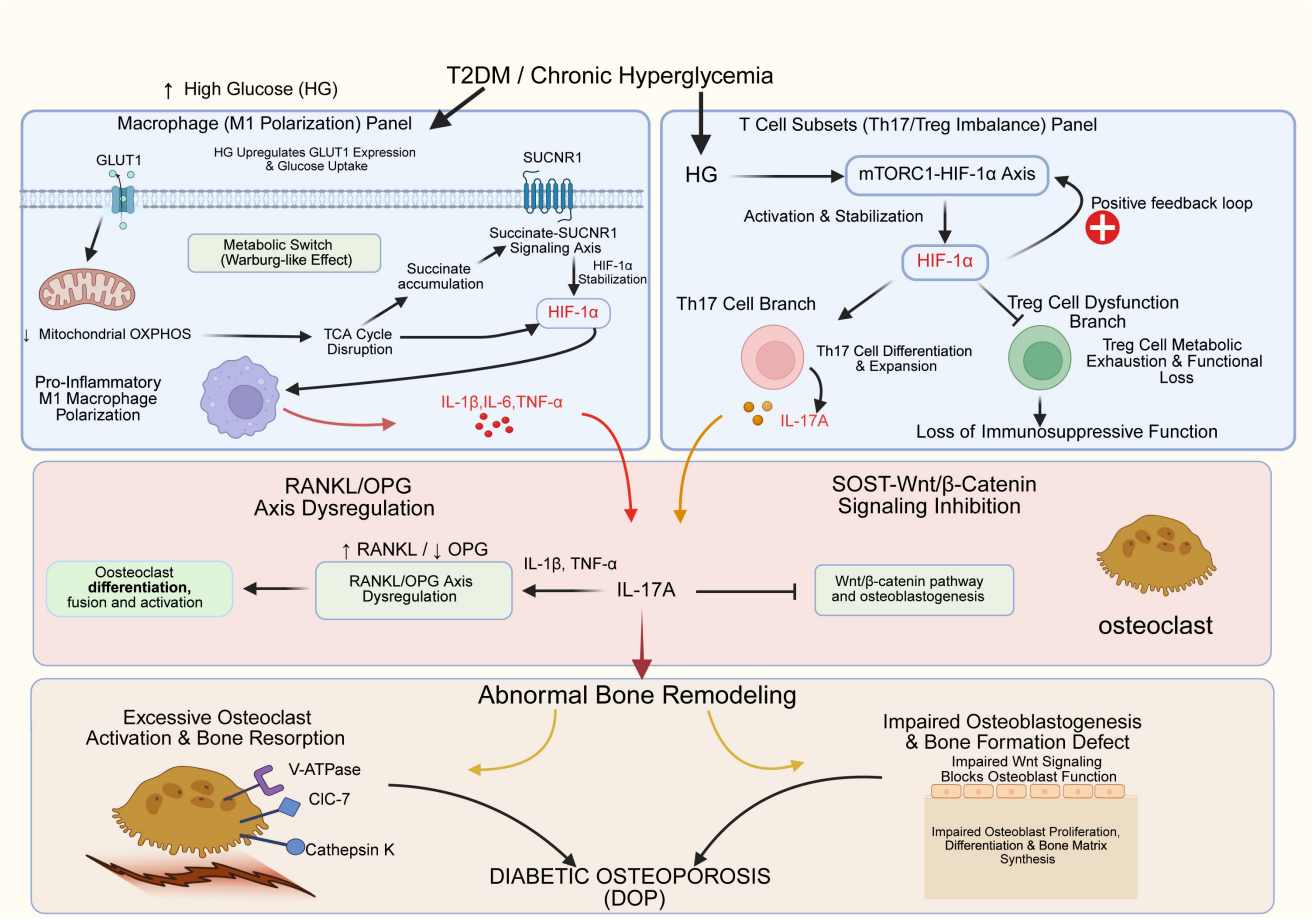
Schematic diagram illustrating the core pathogenic mechanisms by which immunometabolic reprogramming of bone marrow-resident immune cells contributes to pathological bone destruction in type 2 diabetes (T2DM). Chronic hyperglycemia induces metabolic stress and a switch from oxidative phosphorylation (OXPHOS) to aerobic glycolysis (Warburg-like effect) in immune cells via upregulation of glucose transporter 1 (GLUT1). Macrophages undergo M1 polarization driven by the succinate-SUCNR1 signaling axis and HIF-1α stabilization, secreting pro-inflammatory cytokines (IL-1β, IL-6, TNF-α) that promote osteoclast differentiation. Activation of the mTORC1-HIF-1α axis causes Th17/Treg imbalance: enhanced glycolysis expands Th17 cells (secreting IL-17A), while Treg cells undergo metabolic exhaustion and functional loss (some transdifferentiate into pro-inflammatory ex-Tregs). IL-17A disrupts bone homeostasis by tilting the RANKL/OPG ratio toward osteoclastogenesis and promoting sclerostin (SOST)-mediated inhibition of the Wnt/β-catenin pathway (suppressing osteoblastogenesis). Combined excessive osteoclast activation (via V-ATPase/ClC-7 and cathepsin K) and impaired osteoblast function lead to bone microstructural degradation, reduced bone quality, and increased fracture risk. Abbreviations: BMD, bone mineral density; FAO, fatty acid oxidation; HG, high glucose; mTORC1, mechanistic target of rapamycin complex 1; OPG, osteoprotegerin; RANKL, receptor activator of nuclear factor κB ligand; SUCNR1, succinate receptor 1; TCA, tricarboxylic acid; Th17, T helper 17 cells; Treg, regulatory T cells.

Metabolic competition for glucose between Th17 and Treg cells, combined with long-chain fatty acid accumulation and oxidative stress, impairs Treg mitochondrial function and FOXP3 stability (85, 117). A subset of Treg cells transforms into “ex-Tregs,” which lose immunosuppressive activity and acquire pro-inflammatory properties, further amplifying niche inflammation and osteoclastogenesis (119, 120). Critically, metabolically defective Tregs not only lose their intrinsic bone-protective ability but also fail to constrain glycolytic Th17 overactivation, further magnifying Th17-dependent osteoclast formation. Functionally, impaired FAO and defective mitochondrial respiration are the primary drivers of Treg failure in the diabetic niche, whereas enhanced glycolytic dominance in Th17 cells creates a metabolically unfavorable microenvironment for immune tolerance (121). This reciprocal metabolic competition exacerbates niche inflammation, reduces bone formation, and increases bone fragility in diabetic animal models. Preclinical evidence supports that restoring the metabolic fitness of Treg cells through AMPK activation can reverse the Th17/Treg imbalance and mitigate diabetic bone loss, validating the therapeutic promise of targeting Treg metabolism (122, 123) (**Figure 1**).

### 3.4 Bidirectional immunometabolic crosstalk between macrophages and Th17/Treg cells

Macrophages and Th17/Treg cells cooperatively shape the bone marrow immune microenvironment and synergistically regulate bone remodeling during diabetes, rather than functioning independently (18, 28, 66, 124). Hyperglycemia-induced macrophage metabolic reprogramming acts as a priming event for niche immune imbalance. Glycolytic hyperactivation, succinate accumulation, and sustained HIF-1α signaling drive macrophages to secrete abundant TNF-α, IL-6, and metabolic alarmins (125, 126). These paracrine factors activate mTORC1-HIF-1α cascades in CD4 T cells, facilitate glycolysis-dependent Th17 differentiation, and inhibit Treg mitochondrial FAO and FOXP3 stability, ultimately shifting the Th17/Treg ratio toward a pro-inflammatory phenotype (75, 88).

Conversely, abnormal Th17/Treg homeostasis reciprocally amplifies macrophage pro-osteoclastogenic activation. The synergistic effect of elevated Th17 activity and defective Treg function plays a decisive role in sustaining macrophage-mediated bone resorption. Th17-derived IL-17A further enhances macrophage GLUT1 expression, succinate-SUCNR1 axis activity, and inflammatory cytokine release, forming a persistent positive feedback loop in the diabetic bone niche (60, 103). Meanwhile, dysfunctional Treg cells produce insufficient TGF-β and IL-10, abrogating endogenous immune suppression and sustaining macrophage-mediated bone catabolism (96, 119).

**Figure 2:**
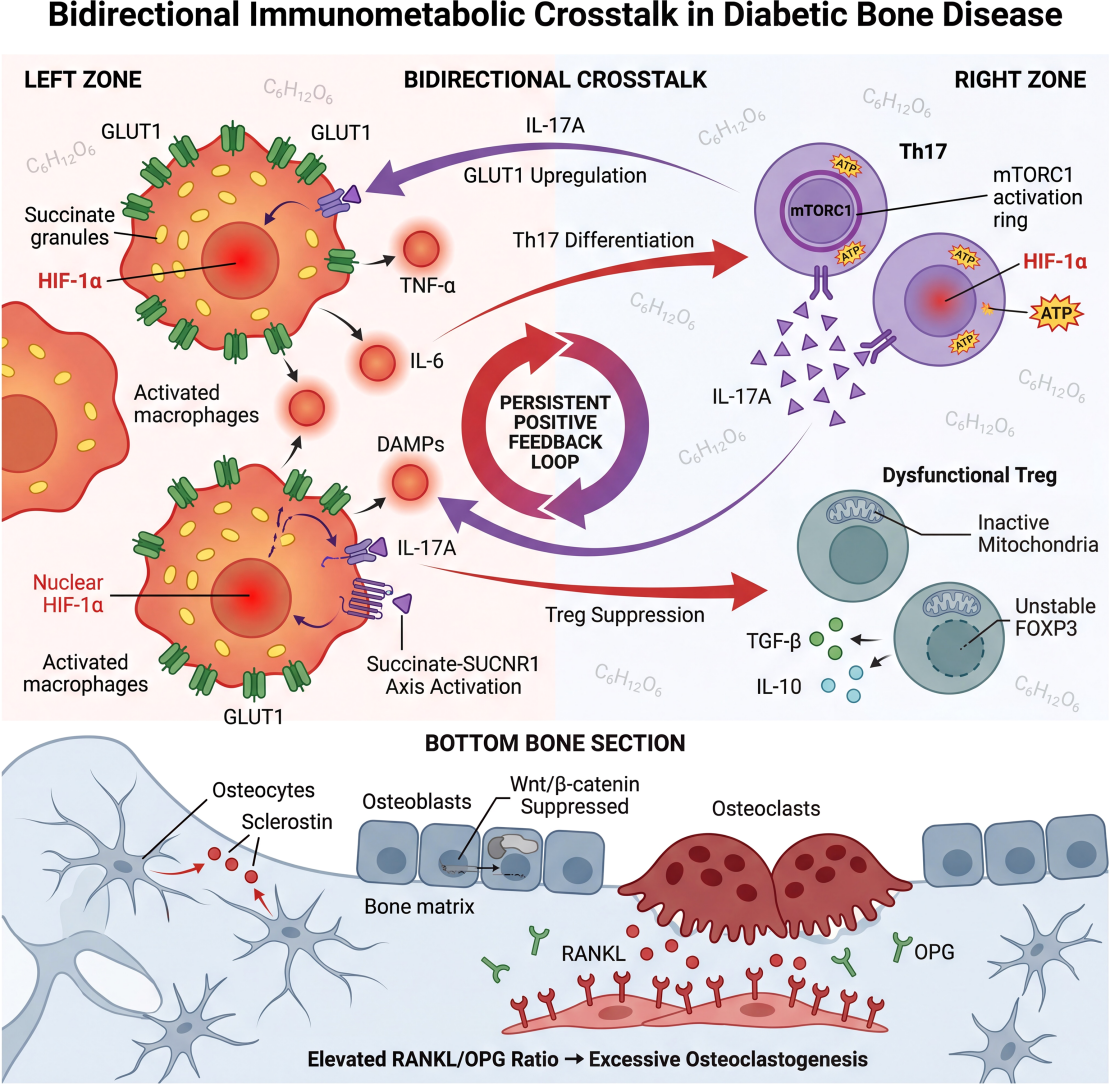
The immunometabolic crosstalk between macrophages and Th17/Treg cells and its regulatory mechanism on bone remodeling in diabetic osteoporosis. Under hyperglycemic conditions, bone marrow macrophages undergo metabolic switch from FAO/OXPHOS to aerobic glycolysis, accompanied by succinate accumulation and persistent HIF-1α activation, leading to the secretion of pro-inflammatory cytokines and metabolites. These paracrine factors facilitate Th17 cell glycolytic differentiation and impair Treg cell metabolic stability, resulting in severe Th17/Treg imbalance. Subsequently, Th17-derived IL-17A reciprocally amplifies macrophage pro-inflammatory activation, forming a sustained immune crosstalk loop. The converging inflammatory signals collectively disrupt skeletal homeostasis via dual regulation of the RANKL/OPG axis and Wnt/β-catenin pathway, ultimately inducing suppressed osteoblastogenesis, excessive osteoclastogenesis, bone microstructural degradation, and diabetic bone fragility. ATP, adenosine triphosphate; C_6_H_12_O_6_, glucose; DAMPs, damage-associated molecular patterns; FOXP3, forkhead box P3; GLUT1, glucose transporter 1; HIF-1α, hypoxia-inducible factor-1α; IL-6, interleukin-6; IL-10, interleukin-10; IL-17A, interleukin-17A; mTORC1, mechanistic target of rapamycin complex 1; OPG, osteoprotegerin; RANKL, receptor activator of nuclear factor κB ligand; SUCNR1, succinate receptor 1; TGF-β, transforming growth factor-β; TNF-α, tumor necrosis factor-α; Wnt/β-catenin, wingless/integrated/β-catenin signaling pathway.

Mechanistically, this bidirectional immunometabolic crosstalk converges on two canonical bone remodeling pathways to trigger DOP pathological progression. Combined pro-inflammatory cytokines markedly elevate the RANKL/OPG ratio to promote excessive osteoclastogenesis and bone resorption (52, 85). In parallel, immune inflammation stimulates osteocytic sclerostin secretion, suppresses Wnt/β-catenin-mediated osteoblastogenesis and bone matrix mineralization (89, 110). In summary, the interplay between Th17-driven pro-osteoclastogenic inflammation and Treg-derived immune tolerance loss serves as the core immunometabolic mechanism that bridges systemic hyperglycemia and irreversible bone microstructural damage (**Figure 2**).

## 4 Therapeutic frontiers: restoring the immunometabolic landscape

Targeting immunometabolic checkpoints has emerged as a novel strategy for DOP treatment, moving beyond conventional glucose-lowering and anti-resorptive therapies to reverse the pathological immune-metabolic niche.

### 4.1 Classification and mechanism of immunometabolic modulators

Given the critical role of immunometabolic dysregulation in the pathogenesis of DOP, targeting key metabolic checkpoints and immune cell phenotypes provides a promising therapeutic strategy for DOP treatment. Unlike conventional glucose-lowering drugs that mainly focus on glycemic control and traditional anti-osteoporotic agents that merely regulate bone remodeling, immunometabolic modulators can simultaneously correct systemic metabolic disorders and local bone marrow immune imbalance, thereby achieving more comprehensive skeletal protection (127–129). To systematically clarify the therapeutic potential of these agents, we summarize the representative immunometabolic modulators, their core mechanisms of action, skeletal effects, research stages, and relevant references in the following table, providing a theoretical basis for clinical translation and individualized treatment of DOP (**Table 2**).

**Table 2:** Potential immunometabolic modulators for diabetic osteoporosis.

Drug class	Representative drug	Immunometabolic mechanism	Skeletal effect	Research stage	Reference
AMPK Activators	Metformin	Activates AMPK to inhibit mTORC1 signaling, suppresses excessive glycolysis, promotes M2 macrophage polarization and Treg stability, and improves the bone microenvironment.	Increases BMD, improves trabecular bone architecture, and inhibits osteoclast activity.	Clinical - Established (adjuvant use in T2D)	(127)
GLP-1 Receptor Agonists	Liraglutide, Semaglutide	Alleviates systemic low-grade inflammation, regulates Th17/Treg balance via gut-immune-bone axis, inhibits NLRP3 inflammasome	Reduces vertebral/hip fracture risk, promotes osteoblast differentiation, enhances bone formation	Clinical- Advanced Phase III/Approved	(128, 129)
SGLT2 Inhibitors	Dapagliflozin, Empagliflozin	Reduces glucotoxicity, inhibits oxidative stress and NLRP3 activation, normalizes macrophage glycolytic flux	Empagliflozin improves bone quality; dapagliflozin carries elevated fracture risk in elderly/frail patients	Clinical - Approved, with Skeletal Safety Concerns	(130, 131)
Glycolysis Inhibitors	2-Deoxy-D-glucose (2-DG), Shikonin	Blocks GLUT1-mediated glucose uptake, inhibits Th17 activation and HIF-1α stabilization, suppresses pro-inflammatory cytokine secretion	Effective in preclinical models; non-specific toxicity and systemic metabolic disruption remain major concerns	Preclinical - *In vitro*/*in vivo* models	(132, 133)
SUCNR1 Antagonists	Selective antagonists (e.g., Compound 7a)	Blocks succinate-SUCNR1 signaling, inhibits macrophage metabolic reprogramming and osteoclast fusion	Effective in preclinical models; selectivity, off-target effects, and long-term safety require validation	Preclinical - *In vivo* models only	(134)

2-DG, 2-Deoxy-D-glucose; AMPK, Adenosine Monophosphate-Activated Protein Kinase; BMD, Bone Mineral Density; GLP-1, Glucagon-Like Peptide-1; GLUT1, Glucose Transporter 1; HIF-1α, Hypoxia-Inducible Factor-1α; mTORC1, Mechanistic Target of Rapamycin Complex 1; NLRP3, NOD-Like Receptor Pyrin Domain-Containing Protein 3; OPG, Osteoprotegerin; RANKL, Receptor Activator of Nuclear Factor κB Ligand; SGLT2, Sodium-Glucose Cotransporter 2; SUCNR1, Succinate Receptor 1; T2D, Type 2 Diabetes; TGF-β, Transforming Growth Factor-β; TNF-α, Tumor Necrosis Factor-α; Wnt/β-catenin, Wingless/Integrated/β-Catenin Signaling Pathway.

### 4.2 Critical appraisal: feasibility, limitations and safety risks

Current immunometabolic targeted strategies exhibit obvious heterogeneity in translational feasibility, clinical applicability and skeletal safety.

Clinically available agents including AMPK activators, GLP-1 RAs and SGLT2 inhibitors have shown definite bone-protective benefits in T2DM populations, but their clinical application still faces prominent limitations. GLP-1 RAs demonstrate favorable fracture prevention effects and dual regulation of immunometabolism and bone homeostasis; however, long-term medication compliance and economic cost remain practical clinical constraints. SGLT2 inhibitors exert beneficial effects on alleviating glucotoxicity and immune inflammation, yet their skeletal safety remains controversial, with increased fracture risk observed in elderly, frail or malnourished diabetic patients, requiring strict individualized medication stratification (130, 131).

In contrast, glycolysis inhibitors and SUCNR1 antagonists are currently limited to preclinical cell and animal experimental stages (132–134). Although these candidates show potent effects in reversing macrophage/T cell metabolic reprogramming and alleviating diabetic bone loss, there are still unresolved challenges including non-specific organ toxicity, off-target immune suppression, unclear optimal dosage and long-term biosafety. At present, evidence supporting their clinical transformation is still insufficient, and large-scale preclinical toxicity verification and pharmacodynamic optimization are urgently needed before further clinical trial design.

### 4.3 Future prospects for clinical translation

Overall, targeting immunometabolic checkpoints provides a novel holistic intervention paradigm for DOP, which differs from single glycemic control or simple anti-resorptive treatment. Future research should focus on establishing precise patient stratification criteria, exploring low-toxicity selective immunometabolic regulators, and carrying out combined regimens of hypoglycemic agents and immunometabolic modulators, to balance therapeutic efficacy, safety and clinical accessibility for the individualized management of DOP.

## 5 Challenges and future perspectives

Despite significant advances in understanding the immunometabolic mechanisms underlying DOP, its clinical management and translational research still face substantial challenges limiting clinical transformation.

### 5.1 Core clinical and diagnostic challenges

The clinical diagnosis and risk prediction of DOP remain problematic due to inherent defects of inherent defects in conventional assessment tools and widespread patient population heterogeneity. Dual-energy X-ray absorptiometry (DXA)-derived BMD, the gold standard for general osteoporosis diagnosis, exhibits poor fracture predictive efficacy in T2DM patients. Accumulated advanced glycation end products (AGEs) and low-turnover bone lesions in diabetic bone tissue lead to normal or elevated BMD accompanied by deteriorated bone microarchitecture, causing widespread DOP underdiagnosis (6, 135). The Fracture Risk Assessment Tool (FRAX) ignores diabetes-specific metabolic disorders and immune inflammation, persistently underestimating fragility fracture risk in T2DM cohorts (1, 135). Although trabecular bone score (TBS) can partially reflect trabecular deterioration, it cannot distinguish immunometabolism-driven pathological bone damage from age-related bone degeneration (7, 136). While high-resolution peripheral quantitative computed tomography (HRpQCT) achieves high-precision microstructural detection, its high cost restricts large-scale clinical promotion (6, 137).

Moreover, the heterogeneity of diabetic patients in age, disease duration, glycemic variability, and comorbidities hinders the establishment of unified DOP diagnostic criteria (15, 25, 71).

### 5.2 Key basic and translational research gaps

In addition to clinical diagnostic limitations, multiple unresolved research bottlenecks hinder the translational progress of DOP immunometabolic investigations. First, the spatial heterogeneity of bone marrow immunometabolism is poorly characterized. Current bulk sequencing-based studies fail to elaborate localized cell-cell crosstalk between immune cells and osteolineage cells, which drives focal trabecular loss and asymmetric bone damage (46, 47). Second, the gut-immune-bone axis regulatory mechanism in diabetic skeletal injury remains elusive. How diabetic intestinal dysbiosis and microbial metabolites remodel bone marrow immune cell metabolism and Th17/Treg homeostasis requires further systematic verification (27, 33). Third, mitochondrial quality control mechanisms (mitophagy, mitochondrial fusion/fission) in immune metabolic reprogramming are insufficiently explored, restricting the discovery of novel targeted targets (10, 24, 80). Fourth, alidated, standardized blood-based immunometabolic biomarkers for DOP clinical screening are lacking (51, 99, 113). Fifth, current artificial intelligence (AI) auxiliary models rely on single-modal clinical data, lacking multi-source data fusion capability, resulting in limited clinical prediction accuracy for DOP (65, 82).

### 5.3 Clinical implications

The immunometabolic dysfunction-driven bone remodeling disorder defined in this review delivers novel mechanistic and clinical insights to advance precision DOP management. Traditional DOP clinical strategies primarily rely on glycemic control and general anti-osteoporotic drugs, which fail to target the root immune-metabolic niche disorder, resulting in limited efficacy in preventing diabetic fragility fractures (14, 15, 137). Collectively, macrophage glycolytic hyperactivation, HIF-1α cascade overactivation, and aberrant Th17/Treg immune crosstalk constitute the core pathological framework of DOP (30, 88, 113). Corresponding clinically mature hypoglycemic agents (GLP-1 RAs, SGLT2 inhibitors) and emerging immunometabolic modulators can reverse niche inflammation, correct unbalanced bone resorption/formation, and improve bone microarchitecture (128, 130).

Clinically, integrated detection of circulating immunometabolic biomarkers (succinate, HIF-1α, IL-17A) combined with conventional imaging tools (DXA, TBS, HRpQCT) enables early screening and individualized fracture risk stratification for T2DM patients (51, 111, 135). Targeted immunometabolic intervention, combined with standardized bone assessment, can break the normal BOP but high fracture risk diagnostic dilemma of DOP, optimize clinical medication regimens, and reduce refractory diabetic fragility fracture incidence (6, 107).

### 5.4 Structured future translational perspectives

To address these challenges, future research should focus on four core directions. First, establish standardized immunometabolic profiling protocols and develop endotype-specific diagnostic criteria for DOP based on multicenter, large-cohort studies, integrating individual patient characteristics to enhance clinical applicability (15, 20, 74).

Second, leverage advanced technologies such as spatial transcriptomics, single-cell multi-omics, and intravital imaging to dissect the spatial heterogeneity of bone marrow immunometabolism, clarifying the crosstalk between different cell types in the bone marrow niche (46, 47). For representative bone and hematopoietic research examples, previous hematopoietic niche studies have verified that spatially localized pro-inflammatory macrophages in the bone marrow induce regional bone resorption and inhibit osteoblast differentiation, which is significantly amplified under hyperglycemic conditions (45, 138). Translating these findings to DOP, targeted spatial immunometabolic profiling can precisely locate localized inflammatory and metabolic dysregulation in diabetic bone marrow, explaining the focal trabecular loss and asymmetric microstructural damage that cannot be captured by global BMD detection, and laying a foundation for site-specific targeted anti-fracture therapy (139).

Third, explore the gut-immune-bone axis in depth, investigating how microbiota-derived metabolites regulate immune cell metabolic reprogramming and developing microbiota-targeted interventions to restore skeletal immune homeostasis (27, 33). Specific hematological and skeletal research has demonstrated that gut microbial short-chain fatty acids can modulate bone marrow Treg/Th17 balance and suppress osteoclastic overactivation, while diabetic intestinal flora disturbance disrupts this homeostatic regulatory pathway (140, 141). Extrapolating to DOP pathogenesis, restoring gut microbial homeostasis and supplementing core protective microbiota metabolites can reverse diabetes-induced skeletal immune metabolic disorder, serving as a novel non-invasive adjuvant strategy for DOP prevention and treatment (45, 142).

Fourth, vigorously promote the integration of AI technology with clinical platforms, developing multimodal AI models that combine biomarkers, endoscopic imaging, and clinical medical records to realize real-time risk assessment and early warning of DOP (65, 74, 82). As validated in existing bone disease research, multimodal AI models integrating serological biomarkers and bone imaging parameters achieve superior fracture prediction accuracy compared with single-modal tools (65, 82). For DOP clinical translation, customized AI algorithms can fuse T2DM-specific indicators (glycemic variability, disease duration) and immunometabolic signatures to correct the inherent risk underestimation of traditional fracture prediction systems, achieving individualized and precise DOP risk stratification for diabetic populations (143, 144). Additionally, longitudinal studies should be conducted to clarify the dynamic changes of immunometabolic biomarkers during treatment, identifying key monitoring nodes to guide clinical treatment adjustment (25, 27).

## 6 Conclusion

DOP is a complex, niche-centric immunometabolic disease resulting from the convergence of systemic metabolic derangement and localized bone marrow immune dysfunction, which underlies the diabetic bone paradox (normal/elevated BMD with increased fracture risk). Chronic hyperglycemia induces metabolic reprogramming of bone marrow-resident immune cells from efficient OXPHOS to pro-inflammatory aerobic glycolysis, disrupting bone resorption-formation balance.

The mTORC1-HIF-1α glycolytic cascade, succinate-SUCNR1 mitochondrial metabolic signaling, and reciprocal Th17/Treg immune imbalance constitute the core triple pathological axes of DOP, dysregulated immune metabolic crosstalk between macrophages and CD4 T cells collaboratively elevates the RANKL/OPG ratio and suppresses Wnt/β-catenin osteogenic signaling, driving excessive osteoclastogenesis and insufficient bone formation (**Figure 2**).

Supported by the above mechanistic framework, targeted immunometabolic intervention strategies covering clinical mature drugs and preclinical candidate agents provide novel therapeutic opportunities for DOP (**Table 2**). Combined with multi-index integrated clinical diagnosis and multimodal AI auxiliary prediction, immunometabolic targeted therapy can effectively solve the clinical dilemma of DOP underdiagnosis and refractory bone loss.

Despite advances, challenges remain in DOP clinical management and translational research. The lack of standardized immunometabolic profiling criteria, insufficient clarification of niche spatial heterogeneity, and limited clinical verification of immune-targeted agents severely hinder the precision treatment of DOP. Future research on spatial immunometabolism, the gut-immune-bone axis, mitochondrial quality control, and multimodal AI integration is critical to overcoming these barriers, with the goal of translating mechanistic insights into precision care to improve DOP management and patient quality of life. Collectively, this review provides a differentiated and comprehensive immunometabolic perspective to interpret DOP pathogenesis, bridges the translational gap between basic immunometabolic research and clinical DOP treatment, and offers novel actionable targets and research directions for future preclinical exploration and individualized clinical intervention.

## Glossary

2-DG: 2-Deoxy-D-glucose
AMPK: Adenosine Monophosphate-Activated Protein Kinase
AI: Artificial Intelligence
BMD: Bone Mineral Density
CD4⁺T: Cluster of Differentiation 4 Positive T Cells
DOP: Diabetic Osteoporosis
DXA: Dual-Energy X-Ray Absorptiometry
FAO: Fatty Acid Oxidation
FOXP3: Forkhead Box P3
GLP-1: Glucagon-Like Peptide-1
GLUT1: Glucose Transporter 1
HIF-1α: Hypoxia-Inducible Factor-1α
HG: High Glucose
IL-10: Interleukin-10
IL-17A: Interleukin-17A
IL-1β: Interleukin-1β
IL-6: Interleukin-6
mTORC1: Mechanistic Target of Rapamycin Complex 1
NLRP3: NOD-Like Receptor Pyrin Domain-Containing Protein 3
OPG: Osteoprotegerin
OXPHOS: Oxidative Phosphorylation
RANK: Receptor Activator of Nuclear Factor-κB
RANKL: Receptor Activator of Nuclear Factor-κB Ligand
RORγt: Retinoic Acid-Related Orphan Receptor Gamma T
ROS: Reactive Oxygen Species
SGLT2: Sodium-Glucose Cotransporter 2
SOST: Sclerostin
SUCNR1: Succinate Receptor 1
T2DM: Type 2 Diabetes Mellitus
TCA: Tricarboxylic Acid
TGF-β: Transforming Growth Factor-β
Th17: T Helper 17 Cells
TNF-α: Tumor Necrosis Factor-α
Treg: Regulatory T Cells
V-ATPase: Vacuolar Adenosine Triphosphatase.
